# Cost of dengue and other febrile illnesses to households in rural Cambodia: a prospective community-based case-control study

**DOI:** 10.1186/1471-2458-9-155

**Published:** 2009-05-27

**Authors:** Rekol Huy, Ole Wichmann, Mark Beatty, Chantha Ngan, Socheat Duong, Harold S Margolis, Sirenda Vong

**Affiliations:** 1 National Dengue Control Program, National Center for Parasitological, Entomology and Malaria Control, Ministry of Health, 372 Blvd. Monivong, Phnom Penh, Cambodia; 2 Pediatric Dengue Vaccine Initiative (PDVI), International Vaccine Institute, SNU Research Park, San 4-8 Bongcheon-7 dong, Seoul, 151-919, South Korea; 3 Epidemiology and Public Health Unit, Institut Pasteur in Cambodia, 5 Bld Monivong – POB 983, Phnom Penh, Cambodia

## Abstract

**Background:**

The average annual reported dengue incidence in Cambodia is 3.3/1,000 among children < 15 years of age (2002–2007). To estimate the economic burden of dengue, accurate cost-of-illness data are essential. We conducted a prospective, community-based, matched case-control study to assess the cost and impact of an episode of dengue fever and other febrile illness on households in rural Cambodia.

**Methods:**

In 2006, active fever surveillance was conducted among a cohort of 6,694 children aged ≤ 15 years in 16 villages in Kampong Cham province, Cambodia. Subsequently, a case-control study was performed by individually assigning one non-dengue febrile control from the cohort to each laboratory-confirmed dengue case. Parents of cases and controls were interviewed using a standardized questionnaire to determine household-level, illness-related expenditures for medical and non-medical costs, and estimated income loss (see Additional file 1). The household socio-economic status was determined and its possible association with health seeking behaviour and the ability to pay for the costs of a febrile illness.

**Results:**

Between September and November 2006, a total of 60 household heads were interviewed: 30 with dengue-positive and 30 with dengue-negative febrile children. Mean total dengue-related costs did not differ from those of other febrile illnesses (31.5 vs. 27.2 US$, p = 0.44). Hospitalization almost tripled the costs of dengue (from 14.3 to 40.1 US$) and doubled the costs of other febrile illnesses (from 17.0 to 36.2 US$). To finance the cost of a febrile illness, 67% of households incurred an average debt of 23.5 US$ and higher debt was associated with hospitalization compared to outpatient treatment (US$ 23.1 vs. US$ 4.5, p < 0.001). These costs compared to an average one-week expenditure on food of US$ 9.5 per household (range 2.5–21.3). In multivariate analysis, higher socio-economic status (odds ratio [OR] 4.4; 95% confidence interval [CI] 1.4–13.2), duration of fever (OR 2.1; 95%CI 1.3–3.5), and age (OR 0.8; 95%CI 0.7–0.9) were independently associated with hospitalization.

**Conclusion:**

In Cambodia, dengue and other febrile illnesses pose a financial burden to households. A possible reason for a lower rate of hospitalization among children from poor households could be the burden of higher illness-related costs and debts.

## Background

Dengue is a major cause of morbidity and mortality in most tropical and subtropical regions of the world, especially in Southeast Asia. Dengue virus (DENV) infection can be asymptomatic or may manifest as an acute febrile illness (dengue fever) with headache and severe myalgias [1]. A small proportion of persons with dengue fever develop severe illness with symptoms of vascular leakage, which usually requires hospitalization and careful fluid management [1]. An estimated 50–100 million individuals are infected with DENV every year, resulting in up to 500,000 dengue patients being hospitalized in some years [2].

Dengue has been reported as a public health problem in Cambodia since 1962. In Cambodia only hospitalized dengue cases < 15 years of age are reported to the Ministry of Health, outbreaks have occurred every two to four years, with a high incidence of reported disease during the inter-epidemic period [3]. The largest ever recorded outbreak occurred in 2007 with 39,851 reported cases and 407 reported deaths (case fatality 1.02%) [Ministry of Health, unpublished data]. Between 2002 and 2007, the average reported incidence of dengue among children < 15 years of age was 3.3/1,000. It can be assumed, however, that this is a considerable underestimation, since studies in neighbouring countries have demonstrated that a substantial proportion of dengue-infected children are not treated in hospitals [4].

Cambodia, located in Southeast Asia, has a population of 13 million, with 40% less than 15 years of age. The country is divided into 24 provinces, 183 districts, 1,623 communes, and 13,408 villages. Cambodia is one of the poorest countries in Asia. In 2004, 84% of the total population was living in rural areas and of those 39% ranked below the national poverty line [5]. Cambodian national poverty lines range from US dollar (US$) 0.43 per capita per day in rural areas to US$ 0.58 in the capital of Phnom Penh [5].

On a household level, it has been reported that any severe illness can cause a reduction in household labour supply, income, and an increase in expenditures [5]. This is particularly important in Cambodia because 90% of total health expenditures are estimated to be out-of-pocket [6]. We conducted a study in a rural area of Cambodia to estimate the cost and impact of an episode of dengue fever and other febrile illness on households. We also determined the socio-economic status of households and assessed its possible association with health seeking behaviour and the ability to pay for the direct medical costs of a febrile illness.

## Methods

From May to November 2006, an active community-based surveillance study was conducted in 16 villages located in 3 districts of Kampong Cham province, Cambodia. The objective was to estimate the seasonal incidence of dengue and the efficiency of the existing national dengue surveillance system to collect case information. During the study period a cohort of 6,694 children aged ≤ 15 years was visited on a weekly basis and blood samples (acute and convalescent) were collected from every child with a fever episode of ≥ 48 hours after obtaining informed consent. Parents were interviewed regarding disease-specific symptoms and health seeking behaviour. Blood samples of all febrile children were tested at the Institut Pasteur in Cambodia for IgM anti-DENV by MAC-ELISA, DENV ribonucleic acid by reverse-transcriptase polymerase chain reaction (RT-PCR) and DENV by cell culture as described previously [7]. An acute dengue case was defined as a person with fever ≥ 38°C for 2 days or longer, and either detection of DENV by RT-PCR or cell culture, or IgM anti-DENV in acute and/or convalescent serum samples.

Within the framework of the surveillance study, a case-control study was conducted between September and December 2006 to compare dengue-specific costs and health seeking behaviours with those of non-dengue febrile illness. One non-dengue fever control was assigned to each acute dengue case; each was recruited from the surveillance study using diagnostic laboratory results. Cases and controls were individually matched by village, age-group (0–4, 5–9, 10–15 years), and date of symptom onset (± 7 days). For controls no attempt was made to determine the aetiology of the fever. Parents or legal guardians of cases and controls were interviewed approximately 1–2 months after recovery from the illness by two trained interviewers after obtaining written informed consent. All data were collected during face to face interviews using a standardized closed-ended questionnaire. The collected variables included demographic information, disease duration and symptoms, perception of health status, health seeking behaviours, care received by the child, indirect and direct costs for parents and other household members, financing of disease-related costs, and housing and food-related indicators (for example, weekly expenditures on food and number of meat-containing meals per week, based on figures from the week prior to interview). In addition, interviewees were asked to rate their perception of the general health of their child during the illness on a four point scale from good to very bad. A two-day training session was held for the four study interviewers in Kampong Cham City and the questionnaire was piloted and validated during this session. The study was approved by the Institutional Review Board of the International Vaccine Institute (#IRB 2006-012) and by the Ethics Committee of the Ministry of Health, Cambodia.

Data were double-entered into EpiInfo (version 3.2, CDC, Atlanta, GA) and analyzed with STATA (Intercooled STATA 9.0 for Windows, StataCorp LP, College Station, TX, USA). Demographic and socio-economic characteristics of cases and controls were compared using McNemar's χ^2^-test for categorical variables and paired t-test for continuous variables. A p-value < 0.05 was considered statistically significant. Disease-specific costs (dengue vs. non-dengue) were compared using a paired t-test or a conditional logistic regression model and the Wald χ^2^-test. The mean and standard deviation (SD) of direct medical costs per febrile patient was calculated according to the patients' estimated out-of-pocket payments for both inpatient and outpatient medical services. Direct non-medical costs included out-of-pocket payments for transportation, food, lodging associated with seeking or obtaining medical care, as well as for household members visiting the sick child at the hospital.

For each household member, indirect costs consisted of: a) income loss due to days of work lost when caring for the child and b) costs to pay somebody to do this work (for example, farmers who needed to take care of their field), which amounted to approximately US$ 1.25 or 5,000 Riels (i.e. the Cambodian currency) per day based on the interviews. If reimbursements were paid to the household by a health equity funds, the amount was subtracted from the direct medical costs of that particular household. Health equity funds have been implemented in Cambodia to increase access for the poor to quality health service by exempting poor patients from paying hospital fees. In the case of the Kampong Cham hospital, other costs such as travel were also reimbursed to the poorest patients. All direct and indirect costs are expressed in 2006 US$ based on 4,000 Riels to 1 US$ currency exchange rate.

We categorized the economic status of each household using a score derived from the following factors: a) average food expenditure per person in the household per week (0–4,999 Riels = 0; 5,000–9,999 Riels = 1, > 9,999 Riels = 2) and b) housing construction (bamboo shed or a house made of improvised mats = 0; solid wood, brick, or metal = 1). A poverty score was the sum of the 2 factors: 0–1 = Very poor; 2 = Poor; 3 = Better-off. To test the association of specific variables (e.g. economic or health status, or demographic factors) with likelihood of hospitalization, a bivariate analysis was performed and all variables with a p-value of ≤ 0.25 were included in a logistic regression model using stepwise backward removal.

## Results

During 2006, a total of 89 patients in the cohort study were found to have a febrile illness that was laboratory-confirmed dengue (incidence 13/1,000). The parents of the last 30 dengue cases were contacted and all agreed to participate in the case-control study. The onset of fever in these 30 cases occurred between the 2^nd ^of July and 22^nd ^of October 2006. Thirty febrile but dengue-negative controls were individually matched to these cases. Their parents were contacted and all agreed to be interviewed. For cases and controls, the mean (SD) duration between disease and interview was 72 (24) days. All 60 febrile illnesses took place in different households for which the mean number of members was six (SD 1.8, range 3–10). There were no differences between cases and controls regarding demographic and economic characteristics (Table 1). However, dengue cases tended to occur less often in households with a high educational status of the father (i.e. at least high school education) when compared to non-dengue febrile illnesses (26% vs. 58%, p = 0.07) (Table 1).

**Table 1:** Demographic characteristic of the study population by disease-group, Kampong Cham Province, Cambodia, 2006

	**Dengue-cases, N (%)**	**Non-dengue controls, N (%)**	**Total N (%)**	P-value*
**Total number**	30 (100)	30 (100)	60 (100)	
**Age-group**				
0–4	12 (40)	12 (40)	24 (40)	
5–9	10 (33)	10 (33)	20 (33)	
10–14	8 (27)	8 (27)	16 (27)	1.00
**Sex**				
Male	19 (63)	15 (50)	34 (57)	
Female	11 (37)	15 (50)	26 (43)	0.32
**Households with > 5 members**	12 (40)	13 (43)	25 (42)	0.72
**Father with at least high-school education**	7 (26)	15 (58)	22 (42)	0.07
**Socio-economic status**				
Very poor	15 (50)	10 (33)	25 (42)	
Poor	10 (33)	14 (47)	24 (40)	
Better-off	5 (17)	6 (20)	11 (18)	0.18

*McNemar's or Wald χ^2 ^test

### Illness presentation, impact on households, and health seeking behaviour

Children were febrile for 7.3 days on average (median 6, range 4–21) including 3.3 days on average (median 3, range 1–8) during which they felt bad or very bad. There were no significant differences between dengue and non-dengue febrile illnesses (Table 2). Hospitalized cases had a greater mean number of fever days (7.9 vs. 6.2 days, p = 0.01) and were thought to feel bad or very bad more frequently than non-hospitalized cases (67% vs. 52%, p = 0.26). Although not significantly different, dengue cases were hospitalized more frequently than children with non-dengue febrile illnesses (controls) (67% vs. 53%; p = 0.16).

**Table 2:** Illness duration, perceived health status during the illness, and hospitalization by disease-group, Kampong Cham Province, Cambodia, 2006

	**Dengue cases (n = 30)**	**Non-dengue controls (n = 30)**	P-value*
**Duration of fever episode (days), mean (SD)**	7.5 (1.8)	7.1 (3.2)	0.59
**Health status reported as bad or very bad**			
Prior to the illness episode, n (%)	0 (0)	0 (0)	1.00
During the illness episode, n (%)	14 (50)	19 (63)	0.37
Total days, mean (SD)	3.3 (1.5)	3.3 (1.2)	0.90
**Hospitalization, n (%)**	20 (67)	16 (53)	0.16
Duration (days), mean (SD)	4.8 (1.6)	4.8 (1.9)	1.00

*McNemar's chi-squared test for categorical and paired t-test for continuous data

Of the 60 children, 15 (25%) attended school and 12 missed school due to the febrile illness for an average of 8.1 days (SD 1.3, range 2–15 days). During the illness a household devoted a mean (SD) of 11.4 (0.9) days taking care of the sick child, which resulted in a mean (SD) of 8.3 (0.73) days of lost work among household members. There was no significant difference in the amount of time lost between households with dengue and non-dengue febrile illnesses. Within the study population, 25 (42%) febrile children were taken to public, 14 (23%) private, 18 (30%) both public and private healthcare facilities and no care was sought for 3 (5%) children. A total of 36 (60%) febrile children were hospitalized. Of these, only 11 (31%) consulted an outpatient facility before presenting at the hospital. The average non-hospitalized febrile child had 1.3 (SD 0.16) outpatient facility contacts; 42% had at least 2 contacts. There was no difference in the number of outpatient visits between children with dengue and non-dengue febrile illnesses.

### Costs and financing of the febrile illness

The average total cost of a laboratory-confirmed dengue illness to the household was US$ 31.5 (range US$ 0–89). The average total cost of a non-dengue febrile illness was US$ 27.2, which was not significantly different (Table 3). The direct medical costs accounted for 50% and 40% of the total illness cost for dengue and non-dengue illnesses, respectively. Average direct medical costs per hospitalized and non-hospitalized dengue case were US$ 19.9 (range 0–60) and 6.6 (range 0–38), respectively. The average total cost per hospitalized dengue and non-dengue illness was similar at US$ 40.1 and US$ 36.2, respectively (Table 4). Among hospitalized dengue cases, the total cost from using private healthcare facilities was significantly higher than that of public healthcare facilities (US$ 55.4 vs. US$ 31.2, p = 0.03). The average cost per outpatient visit was US$ 12, and although not statistically significant, the average total cost per non-hospitalized dengue illness was higher from private healthcare provider compared to public providers (US$ 17.9 vs. US$ 10.8; p = 0.63).

**Table 3:** Costs (in US$) to households per febrile episode by disease-group, Kampong Cham Province, Cambodia, 2006

	**Dengue cases (n = 30)**		**Non-dengue controls (n = 30)**		
		
	Mean	Median (range)	Mean	Median (range)	P-value*
Direct medical costs	15.5	10.3 (0–60)	11.1	9.0 (0–50)	0.33
Direct non-medical cost	6.6	0.6 (0–29)	2.4	0 (0–25)	0.07
Indirect costs	9.5	8.8 (0–30)	13.9	10.3 (0–40)	0.02

**Total costs**	**31.5**	**31.3 (0–89)**	**27.2**	**25.8 (0–75)**	**0.44**

*Paired t-test

**Table 4:** Total costs (in US$) to households per average febrile illness by type of health-care provider, Kampong Cham Province, Cambodia, 2006

	**Dengue cases**		**Non-Dengue controls**		P-value*
		
	Mean	Median (range)	Mean	Median (range)	
**Total cases**					
Hospitalized (n = 36)	40.1	44.9 (0–89)	36.2	36.8 (0–75)	0.48
Non-hospitalized (n = 24)	14.3**	10.4 (0–56)	17.0**	13.8 (0–44)	0.34
**Hospitalized cases**					
Public provider only (n = 23)	31.2	37.3 (0–77)	32.7	38.1 (0–54)	0.3
Private provider only (n = 12)	55.4**	56.5 (46–61)	40.8	35.0 (9–75)	1.0
**Non-hospitalized cases**					
Public provider only (n = 6)	10.8	13.6 (0.3–19)	15.8	7.0 (1–39)	1.0
Private provider only (n = 11)	17.9	7.3 (2–56)	20.3	18.1 (8–44)	0.46

*Matched analysis using conditional logistic regression

**Significant difference (p < 0.05) between the two sub-groups in the column, independent t-test

To finance the febrile illnesses, 40 (67%) households incurred an average debt of US$ 23.5 (range: US$ 0.5–50) by borrowing money from friends, neighbours, or local money lenders. This was more than double the average amount households spent on food in 2 weeks (mean US$ 9.5 per week prior to interview). Hospitalization significantly increased incurred debt from US$ 4.5 (outpatient) to US$ 23.1 (p < 0.0001). However, there was no difference in the amount of debt incurred per dengue and non-dengue illness (US$ 17 vs. US$ 14.3, p = 0.54) or whether care occurred in the public or private sector (US$ 20.3 vs. US$ 11.4, p = 0.09).

Other sources to finance the febrile illness included use of current income (55%), selling assets (18%) and savings (7%). Eight (13%) households were reimbursed (mean: US$ 16.3; range: 11.3–35) for medical expenditures at the public hospital through a health equity fund of the Belgium Technical Cooperation. Six (60%) of the 10 households classified as very poor and who had a child hospitalized received reimbursements. For these six cases, the total direct medical and non-medical costs for hospitalization were reduced to a mean of US$ 3.5 (range 0–7.5), and the average total costs per hospitalized case in this subgroup was US$ 13.7 (all six used the public hospital). Without considering these reimbursements, the average total costs per hospitalized case in a public hospital was US$ 35.8 (median: US$ 39.5; range: 6–77).

### Association of poverty with health seeking behaviour and financing

Based on the poverty score, 25 (42%) households belonged to the very poor, 24 (40%) to the poor, and 11 (18%) to the better-off category (Table 1). Being in the better-off category was significantly associated with higher (i.e. at least high school attendance) father's education (27%, 45%, and 64%, p = 0.003). Other measures of socio-economic status were that 34 (57%) households had a television, 9 (15%) a DVD-player, and 6 (10%) owned a mobile phone. The majority (93%) of households were living in a one-room dwelling, which was usually made of bamboo and mats (n = 23, 38%), or of wood (n = 32, 53%). Lighting was most often a kerosene lamp (n = 28, 47%) or from battery-generated electricity (n = 26, 43%). The average one-week expenditure for food per household during the week prior to interview was US$ 9.5 (range: US$ 2.5–21.3) and per household member US$ 1.75 (range: US$ 0.4–4.4).

In bivariate analysis, poverty (40%, 71%, 82%, p = 0.008) and duration of fever (p = 0.011) but not age of the child (p = 0.23) or perceived health status (bad vs. very bad, p = 0.25) were associated with hospitalization. In multivariate analysis, high poverty score (odds ratio [OR] 4.4; 95% confidence interval [CI] 1.4–13.2), duration of fever (OR 2.1; 95% CI 1.3–3.5), age (OR 0.8; 95% CI 0.7 – 0.9) and incurring debts (OR 12.8; 95% CI 2.1 – 77.7) remained independently associated with hospitalization, but not the perceived child's health status, sex, and educational background of the parents (p > 0.2). For all households, there was a trend that the very poor and poor borrowed money more frequently to finance the febrile illness than better-off households, and this trend was most evident among households with non-hospitalized children (60%, 29%, 0%, p = 0.08) than among households with hospitalized children (80%, 88%, 66%, p = 0.49).

## Discussion

The present study showed that dengue and other febrile illnesses have a substantial financial impact on households in a rural province of Cambodia. Direct medical costs accounted for about 50% of the economic impact; the remaining 50% included non-medical costs of caring for the ill child and actual loss of income due to work loss or the need to pay someone to take care of their rice fields. To pay these costs, two-thirds of households had to borrow money and 25% had to sell assets or use their savings.

This study assessed the cost of a dengue febrile illness irrespective of whether the person was hospitalized or not. This distinction is particularly important to a country because the greater burden of disease is due to persons cared for as outpatients [4], yet most published dengue cost-of-illness studies have focused on hospitalized patients [6,8-10]. As expected, the total costs to the household increased dramatically if the child was hospitalized, but at US$ 14, the cost of non-hospitalized dengue was still substantial considering that 39% of people in rural Cambodia have a daily income below US$ 0.43 [5].

The total cost of a confirmed dengue illness was not different from a non-dengue febrile illness, a finding that differs from a study in northern Thailand [4]. There the average total cost of a dengue illness (US$ 16.6) was significantly higher than for a non-dengue illness (US$ 9.8) but also the duration of the dengue-related illness was longer than the non-dengue illness and especially the proportion of hospitalization was much higher among dengue patients (33% vs. 1%) [4]. In Thailand, the average cost of a dengue illness was lower compared to that of Cambodia, which may be due to higher hospitalization rate in our study population in Cambodia (67%). However, the cost per hospitalized dengue case in our study was similar to that of hospitalized dengue hemorrhagic fever patients in Thailand (US$ 40 vs. 39) [4].

A study in two villages of Kampong Cham province in 2003 showed the average direct medical and non-medical cost of a hospitalized case of dengue was US$ 34.5, which did not include indirect costs such as loss of income [9]. In Banteay Meanchey, a rural Cambodian province bordering Thailand, the average household expenditure for dengue treatment at private providers was US$ 103 [6]. The Cambodian data suggests that differences in costs-of-illness may exist by type of health service utilized (private or public), and that cost-of-illness assessments should be conducted in multiple sites if possible [11]. Differences might have also occurred due to different methodologies in how data were collected and which -especially indirect- costs were included.

Health equity funds attempt to improve access to health care services for the poorest. A review of four hospital-based health equity funds in Cambodia showed they increased utilization of hospital services by the poorest patients [12]. In our study population, 60% of households classified as very poor received reimbursements through an equity fund if their child was hospitalized for a febrile illness, which reduced the direct cost to less than 7.5 US$ per hospitalized child. Still, a large proportion of our study population incurred debts to finance the febrile illness. Hospitalization was highly associated with higher average debt, and inversely related to poverty. These associations indicate that dengue and other febrile illnesses continue to exacerbate a family's financial burden, and lack of financial resources probably played a role in the decision whether to manage a sick child in the hospital or at home. In addition, a qualitative study performed in the same province suggested that lack of financial recourses lead to delays in help seeking and inappropriate treatment of children with dengue [9,13].

Traditionally economists have relied on reported income and expenditures as the preferred indicator of poverty [5], but recent research has shown this kind of data are often subject to measurement error or systematic reporting biases due to differing interpretations of the questions by respondents [14]. For this reason we created a poverty scale, which combined information on food expenditure with information on the ownership of a selected asset [14], which in our opinion reflected best the socioeconomic status of a household in rural Cambodia. Information on housing was also used by the Cambodian socio-economic survey in 2004 [15]. Although our goal was to only classify our study population and not compare it with other populations, nonetheless, 42% of our study households were classified as very poor, which is almost the same proportion (39%) of Cambodians in rural areas that lived below the national poverty line, as determined by the World Bank in 2004 [5]. Also the observed associations of poverty with the educational level of the father and the magnitude of incurred debts indicated that our poverty score provided a valid assessment tool.

There were several limitations of the study. Its small sample size didn't allow for robust comparisons by type of health care provider once cases were stratified by dengue and non-dengue febrile illness, and hospitalization. The study was conducted in a rural area of a single province, which is more densely populated in comparison to especially the northern provinces but had in 2004 with 35 to 45% an average poverty headcount for rural areas of Cambodia (with the highest in the south-western plain region and the lowest in the coastal provinces) [5]. Main socio-economic and structural differences in the country exist, however, between rural and urban areas [5].

Thus, our study population may be representative of rural Cambodia, where 84% of the total population lives, but not be representative of the entire country. In addition, costs of a dengue episode may vary broadly from one health facility to another depending on how well the health equity funds operate – User-fees exemption for the poor shown in some Cambodian hospitals could worsen inequity [9,16]. Anecdotal reports made us believe that the health equity funds in Kampong Cham hospital has helped increase hospital access for the poor. The study design of matching laboratory-confirmed dengue cases with dengue-negative controls lead to some delay in conducting the interviews, which might have introduced some recall problems. However, recall problems should have occurred for both cases and controls and might have not introduced a bias when comparison them. Last, the strength of the association between poverty score and hospitalization for children may be limited because there were no independent measures of disease severity other than fever duration, which may have affected the decision to hospitalize.

The major strength of the study was that hospitalized and non-hospitalized cases and controls were recruited from a prospective community-based cohort study, and included cases that did not seek medical attention. Importantly, the case-control design allowed for robust comparisons of hospitalized and non-hospitalized cases, and the dengue and non-dengue febrile illnesses.

## Conclusion

In poor communities like those found in rural Cambodia, dengue, like other febrile illnesses, imposes a severe financial hardship on families, particularly if hospitalization is required. Although equity health funds were able to reduce some of the financial burden on these families, poverty was still significantly associated with a lower chance that a child was treated in a hospital.

Dengue is an important cause of febrile illness in Asia and the Americas. To sustain the reduction of the overall burden of dengue, it is recognized that dengue vaccines are needed; hence several vaccine candidates are currently in development [17]. To assess the cost-effectiveness of the potential use of a dengue vaccine but also other control measures, country-specific cost-of-illness data are needed for hospitalized and non-hospitalized cases cared for in the public and private sector.

## Competing interests

The authors declare that they have no competing interests.

## Authors' contributions

RH contributed to the design of the study, conducted the study, participated in statistical analysis and interpretation of data, and manuscript preparation. OW contributed to the statistical analysis, interpretation of findings and manuscript preparation. MB participated in data management and manuscript preparation. CN, DS and HSM contributed to study design and manuscript revisions. SV conceived the study and contributed to data interpretation and manuscript preparation. All authors read and approved the final manuscript.

## Pre-publication history

The pre-publication history for this paper can be accessed here:



## Supplementary Material

Additional File 1 **2006 cost study survey questionnaire, Cambodia**. the questionnaire represents the data collection instrument that was developed and used during the present study. Click here for file
