# Supplementary material for: Cost of dengue and other febrile illnesses to households in rural Cambodia: a prospective community-based case-control study
Source: BMC Public Health. 2009 May 27;9:155. doi: 10.1186/1471-2458-9-155 (PMC2696434; doi:10.1186/1471-2458-9-155)
Supplement: Additional File 1 — 2006 cost study survey questionnaire, Cambodia. the questionnaire represents the data collection instrument that was developed and used during the present study. [file 1471-2458-9-155-S1.pdf]

GENERAL INSTRUCTIONS

The objective of this study is to understand all the relevant events that have happened to your child and any other household member during your [child name]'s fever episode.

PATIENT'S names

Given name	
Family name	

IN ORDER TO PROTECT PATIENT CONFIDENTIALITY, THIS PAGE WILL BE DETACHED BY THE PROJECT MANAGER OR PRINCIPAL INVESTIGATOR AFTER THE INTERVIEW AND BEFORE THE DATA FROM THIS FORM IS ENTERED INTO A DATABASE. FORMS FROM THE SAME PATIENT SHOULD HAVE THE SAME SUBJECT I.D. NUMBER IN ORDER TO MATCH INFORMATION FROM DIFFERENT FORMS WITH THE SAME STUDY SUBJECT.

Please assign in the box the subject I.D. number for the patient (3-digit ID for this study) [e.g. 001, 019, 123]. All forms -interviews of a same patient, should have the SAME SUBJECT I.D. number. Please also write this Subject I.D. number on the top of the next page before detaching this page.

For the interviewer: Fill in the dates of beginning and end of the [child's name]'s fever episode. The period when the child was sick is called Reference Period of the interview.

Sun	Mon	Tue	Wed	Thu	Fri	Sat

The child started with fever on:

dd//mm//yyyy : ....//..................//.................

The interview is expected on (...... days later):

dd//mm//yyyy : ....//..................//.................

Subject I.D. number	

Interviewer name	

A1.	Date of this interview	dd/mm/yyyy	
A2.	When did [child's name]'s start with fever?	dd/mm/yyyy	
A3.	When did [child's name]'s recover completely from his/her fever episode? If not recovered at the time of the interview, leave it blank.	dd/mm/yyyy	

A4.	**Interviewee**	**1.** Father	**2.** Mother	3. Father and Mother	4. Other	

If interviewing other than father of mother, please specify:

A5.	

A. PATIENT DEMOGRAPHICS

☞

Use the name of the child *[child's name]* who was or is sick to ask questions

A6.	What is [***child's name]'s sex?***	**1.** Female **2.** Male	
A7.	How old (in years) was [child's name] at her/his last birthday? (if under age 1, then write "0")	Years	

B. FEVER EPISODE

B1.	How many days was [child's name] sick?	Days	
B2.	At the time of this interview, is [child's name] still experiencing symptoms such as fever?	**1.** Yes **2.** No	

☞

The next two questions are about [child's name]'s overall health.

B3.	How would you evaluate [child's name] 's health before this fever episode?				
	**1.** Very good	**2.** Good	**3.** Average	**4.** Bad	**5.** Very bad

B4.	How would you evaluate [child's name] 's health during his/her fever episode?				
	**1.** Very good	**2.** Good	**3.** Average	**4.** Bad	**5.** Very bad

☞ Think about the total number of days that [child's name] was sick:

B5.	For how many days did [child's name] feel "bad" or "very bad"?

C. CARE RECEIVED BY YOUR CHILD DURING HIS/HER FEVER EPISODE

☞ Think about each time [child's name] was seen by a care provider during his illness, and help us to complete the following table with each care recievd order chronologically.

	Type of provider	Type of care	Out of pocket spending		Any reimbursement of your spending by employer, health insurance, government, etc		Number of days fever (g)
	**1.** Public **2.** Private (a)	**1.** Emergency 2. Hospitalization 3. Health Center 4. Phamacy 5. Healter 6. Ambulatory visit (b)	Amount spent on the medical provider in RIELS ('c)	Amount spent on transportation, meals, etc. In RIELS (d)	**1.** Yes **2.** No (e)	Amount reimbursed in RIELS (f)	
Visit one C1.							
Visit two C2.							
Visit three C3.							
Visit four C4.							
Visit five C5.							

C6	If your child was hospitalized during the illness episode, please report the total number of nights that your child spent in the hospital(s)

D. ILLNESS IMPACT ON HOUSEHOLD MEMBERS

☞ We would like to learn about how [child's name]'s fever episode affected the different members of your family.

D1.	How many members live in the patient's household?	members

☞ We would like to know the age and sex of the different members of your family, their education, if they are studying or working and if they have lost days of schools or work due to [child's name]'s fever episode.

For level of education of each household member, please use the following codes:

**1.** No formal education	**2.** Primary school not completed	**3.** Primary school completed	**4.** High school not completed
**5.** High school completed	**6.** Vocational school	**7.** College or more	

If you or any of your household member is self-employed and lost days of work, please estimate the amount of income lost (if applicable) for work days off while taking care of [child's name].

For "income lost", include monetary values in local currency.

For each household member who spent time caring for [child's name] when he was sick, please report the number of days of care and the average number of hours per day.

Relation to the sick child		CURRENTLY STUDYNG ?	CURRENTLY WORKING ?	CARED FOR PATIENT?

	Age in years (a)	Sex 1. Female 2. Male (b)	Highest education code ('c)	Studying (Y/N) (d)	Days of school absence (e)	Working for pay (Y/N) (f)	Days of work absence (g)	Income lost? (Y/N) (h)	Amount lost in RIELS (i)	Number of days (j)	Average daily number of hours (k)
Sick child D2.										N.A.	N.A.
Father D3.											
Mother D4.											
Grandparent 1 D5.											
Grandparent 2 D6.											
Sibling 1 D7.											
Sibling 2 D8.											
Sibling 3 D9.											
Sibling 4 D10.											
Other 1 D11.											
Other 2 D12.											
Other 3 D13.											

D14.	Was the fever episode of [child's name] during school holidays?	**1.** Yes **2.** No	

E. CHALLENGES DURING THE REFERENCE PERIOD

☞ We would like to learn if [child's name] experienced any of the following situation during the fever episode.

E1.	Did [child's name] receive care in time?	**1.** Yes **2.** No	
E2.	Did [child's name] need medicines?	**1.** Yes **2.** No	

F. FINANCING THE FEVER EPISODE

☞ Which of the following financial sources did you use to pay for any health expenditures related to [child's name]'s fever episode episode?

F1.	Did you use current income of any household member?	**1.** Yes **2.** No	
F2.	Did you use savings (e.g. bank account)?	**1.** Yes **2.** No	
F3.	Did you have to borrow money from family members or friends from outside the household?	**1.** Yes **2.** No	
F4.	If yes, specify the monetary amount in RIELS		
F5.	Did you have to borrow from someone other than a friend or family?	**1.** Yes **2.** No	
F6.	If yes, specify the monetary amount in RIELS		
F7.	Did you have to sell or transfer any household items (e.g. animals) to finance the care of [child's name] ?	**1.** Yes **2.** No	
F8.	If yes, specify the monetary value.		
F9.	Did you receive help in financing the fever episode of [child's name] from any other party (e.g temple)?	**1.** Yes **2.** No	

G. HOUSING AND FOOD RELATED INDICATORS

G1.	How many **rooms** in the dwelling unit are used by the household (other than kitchen, toilet and bathrooms)	rooms	

G2.	What is the primary construction material of the **outer wall** of the housing/dwelling unit occupied by your household?				
	1.Bamboo, Thatch	2.Wood or logs	3.Plywood	4.Concrete, brick, stone	
	5.Galvanized iron or aluminium	6.Fibrous cement	7.Makeshift, salvaged or improvised materials	8. Other (Specify)	

G3.	What are the primary construction material of the **roof** of the housing /dwelling unit occupied by your household?				
	1.Thatch	2.Tiles	3.Fibrous cement	4.Galvanized iron or aluminium	5.Salvaged materials
	6.Mixed but predominantly made of galvanized iron/aluminium, tiles or fibrous cement	7.Mixed but predominantly made of thatch or salvaged materials	8. Concrete	9.Plastic sheet	10.Other
G4.	What is your household's main **source of lighting**?				
	1.Publicly-provided electricity		2.Privately-generated electricity/Generator		3.Battery
	4. Kerosene lamp		5. None		6.Other

FOOD RELATED INDICATORS

G5.	During the last 12 months, did you have a stock of rice that was sufficient for at least one month of household consumption?	**1.** Yes **2.** No
G6.	If yes, when you had your maximum stock of rice, for how many months was it sufficient to feed your household?	months
G7.	How many times in the past 7 days did your household consume big fish, squid, shrimp and prawns, etc. If never, write '0'	times
G8.	How many times in the past 7 days did your household consume other meat (beef, pork, chicken, duck, etc.). If never, write '0'	times
G9.	In the last 12 months, has this household had enough food all days or were there days and weeks with very little or no food so that the household had to starve?	**1.** Yes **2.** No
G10.	How many of the last 52 weeks did the household have so little food that it was starving? Write 0 if less than 1 week	weeks

G11.	In the past 7 days, how much did your household spend on **Food**, including such things as rice, meat, fruits, vegetables, and cooking oils. Include the value of any food that was produced and consumed by the household, and exclude alcohol, tobacco and restaurant meals.

H. HOUSEHOLD ASSETS

☞ How many of the following items does the household own? (Write '0' if none)

H1.	Television	Number	
H2.	Cellphone	Number	
H3.	DVD-Video cassette recorders	Number	
H4.	Refrigerator	Number	
H5.	Fan	Number	
H6.	Generator	Number	
H7.	Bicycle	Number	
H8.	Motocycle	Number	
H9.	Cattle	Number	
H10.	Pigs	Number	
H11.	Chicken/Poultry	Number	

E. LAB RESULTS:

E1.	Dengue virus infection	**1.** Yes **2.** No
